# Conventional and Virtual Reality Mirror Therapies in Upper Obstetric Brachial Palsy: A Randomized Pilot Study

**DOI:** 10.3390/jcm9093021

**Published:** 2020-09-19

**Authors:** Alba Yeves-Lite, Juan Carlos Zuil-Escobar, Carmen Martínez-Cepa, Helena Romay-Barrero, Asunción Ferri-Morales, Rocío Palomo-Carrión

**Affiliations:** 1 San-José Institute Foundation, Avda. de la Hospitalidad, s/n, 28054 Madrid, Spain; albayeves@hotmail.com; 2 Department of Physiotherapy, Faculty of Medicine, CEU-San Pablo University, Urb. Montepríncipe, s/n., 28668 Madrid, Spain; 3 Department of Nursing, Physiotherapy and Occupational Therapy, Faculty of Physiotherapy and Nursing, University of Castilla-La Mancha, 45071 Toledo, Spain; Helena.Romay@uclm.es (H.R.-B.); Asuncion.Ferri@uclm.es (A.F.-M.); Rocio.Palomo@uclm.es (R.P.-C.)

**Keywords:** family, mirror therapy, neonatal brachial plexus palsy, upper extremity, virtual reality

## Abstract

The abilities of children diagnosed with Obstetric Brachial Palsy (OBP) are limited by brachial plexus injuries. Thus, their participation in the community is hindered, which involves a lower quality of life due to worse performance in activities of daily living as a consequence of the functional limitations of the affected upper limb. Conventional Mirror Therapy (Conventional MT) and Virtual Therapy improve the affected upper limb functionality. Therefore, the aim of this study was to compare the effects of Conventional MT and Virtual Reality MT on the spontaneous use of the affected upper limb and quality of life of children with upper Obstetric Brachial Palsy between 6 and 12 years of age. A randomized pilot study was performed. Twelve children were randomly assigned to perform Conventional Mirror Therapy or Virtual Reality Mirror Therapy for four weeks. Ten children completed the treatment. Two assessments (pre/post-intervention) were carried out to assess the spontaneous use of the affected upper limb and the quality of life using the Children’s Hand-use Experience Questionnaire (CHEQ) and the Pediatric Quality of Life Inventory Generic Core Scales (PedsQL TM 4.0), respectively. There was a statistically significant increment in spontaneous use, observed in independent tasks (*p* = 0.02) and in the use of the affected hand with grasp (*p* = 0.04), measured with the CHEQ, for the Virtual Reality MT group. There were no statistically significant changes (*p* > 0.05) for the Conventional MT group in the spontaneous use of the affected upper limb. Regarding the quality of life, statistically significant changes were obtained in the Physical and Health activity categories of the parents’ questionnaire (*p* = 0.03) and in the total score of the children’s questionnaire (*p* = 0.04) in the Virtual Reality MT group, measured using the PedsQL TM 4.0. Statistically significant changes were not obtained for the quality of life in the Conventional MT group. This study suggests that, compared to Conventional MT, Virtual Reality MT would be a home-based therapeutic complement to increase independent bimanual tasks using grasp in the affected upper limb and improve the quality of life of children diagnosed with upper OBP in the age range of 6–12 years.

## 1. Introduction

Obstetric Brachial Palsy (OBP) is defined as a partial or total flaccid paralysis that affects the upper limb of the newborn due to brachial plexus injury occurring in normal delivery, and, more rarely, in cesarean section, often associated with shoulder dystocia [1]. OBP results from injury to the cervical roots C5–C8 and thoracic root T1 [2] with <1% of births. It is a serious complication, whose occurrence increases considerably to 6% in cases of fetuses weighing >4000 g [3,4].

There are different classifications of OBP in terms of anatomical location: upper, intermediate, lower, and total plexus palsy [2]. Upper plexus palsy involves C5, C6, and sometimes C7. Also called Erb’s palsy, it is the most common type of brachial plexus injury (47% of all OBP cases reported) [2,5,6]. It presents with an adducted arm, which is internally rotated at the shoulder. The wrist is flexed, and the fingers are extended, resulting in the characteristic “waiter’s tip” posture. Intermediate plexus palsy involves C7 and sometimes C8 and T1 [1,2,7]. Lower plexus palsy involves C8 and T1 [1,2,8]. Also called Klumpke paralysis, it is very rare, with 2% of all OBP cases reported [2]. The main clinical feature is poor hand grasp, whereas more proximal muscles are intact [1,2]. Total plexus palsy involves C5–C8 and sometimes T1 [1,2], and it is the second most common type of OBP injury [8]. It is the most devastating plexus injury: the infant is left with a clawed hand and a flaccid and insensate arm.

Clinical findings can be classified into four categories, according to Narakas [9,10]: Group I (C5–C6 paralysis of the shoulder and biceps brachii), Group II (C5–C7 paralysis of the shoulder, biceps brachii, and forearm extensors), Group III (C5–T1 complete paralysis of the limb) and Group IV (complete paralysis of the limb with Horner’s syndrome) [9,10].

The right arm is more frequently involved due to the more prevalent left occipitoanterior position at delivery [2,11].

The involvement of muscles (depending on the type of injury) results in permanent contractures, impaired mobility, and muscle weakness. Thus, compromising the spontaneous use of the affected upper limb and the quality of life in children [12]. Since the abilities of these children are limited by brachial plexus injuries, their participation in the community is hindered, which involves a lower quality of life due to worse performance in activities of daily living, as a consequence of the functional limitations of the affected upper limb [13].

Early intervention in OBP is essential for the recovery of the movement and sensitivity of the affected limb. The treatment of choice is reconstructive microsurgery, which is used to establish connections between the nerves of the brachial plexus or through grafts, as well as muscle transfer surgery, through which certain muscles are loosened [14]. One of the most common treatment is conventional physiotherapy with passive mobilizations to prevent shortening, which is performed within the physiological range of each joint to avoid excessive stretching. Strength exercises and active mobilizations are also applied [15]. Kinesiotaping applied in different areas of the affected upper limb to help activate certain muscles, as well as electrostimulation associated with an exercise program, are used in the treatment of Obstetric Brachial Plexus Palsy [15]. Constraint-Induced Movement therapy is one of the most used therapies in this neurological involvement; it consists in the total or partial containment of the healthy upper limb, in order to achieve a greater use of the affected upper limb in activities of daily living [16,17]. However, sensitivity is also affected, thus the treatment of sensory stimulation is important in the integration of the affected limb through tactile and proprioceptive stimuli. Other treatments are more invasive [18]. Botox is also used for shoulder, elbow, and forearm movement imbalance [19], although it must be combined with other therapies, such as conventional physiotherapy [19] or constraint-induced movement therapy, in order to sustain improvements over time [20].

The limitation in functionality implies the need to apply specific therapies to facilitate manual abilities of the affected side, improving its integration into the natural environment of the child. According to the ecological vision of human development, it is crucial to incorporate the principles of therapy into the environment in which the child develops, which is essential for the long-term prevalence of the achieved results [21]. From the ecological point of view, the evolution of the child is understood as a process of progressive differentiation of the activities that he/she carries out, his/her role and his/her interactions with the environment. The interactions and transactions established between the child and the elements of his/her environment are very important, especially with his/her parents [21,22].

Conventional Mirror Therapy (conventional MT) and Virtual Reality Mirror Therapy (Virtual Reality MT) are two therapeutic strategies whose goal is to improve the affected upper limb functionality and the quality of life in different disorders, including OBP, and both can be performed at home to reduce the parental stress and increase the family-child interaction [21].

Mirror therapy (MT) is a rehabilitation strategy based on the repeated use of the mirror illusion (MI). Patients train by looking into a mirror placed along their midline and hiding their defective limb. The observed reflection of the unimpaired limb superimposes itself on the defective one, thus generating the visual illusion of a functional limb. MT was initially devised as a strategy to alleviate phantom limb pain in amputees before being applied as a neurorehabilitation approach for hemiparetic adults after stroke [23]. There is increasing evidence from randomized controlled trials regarding the effectiveness of MT for improving upper limb motor function, activities of daily living and pain, at least as an adjunct to normal rehabilitation for adults after stroke [24]. In children with hemiplegia, a single pilot clinical trial demonstrated that MT may increase the strength of the paretic arm and improve its dynamic function [25]. The mechanism believed to underlie MT is its effect on “learned paralysis”, in which conflicts between motor efferences and reafferent sensory feedbacks impede motor function [23]. Every time a motor command is sent to the paretic limb, the returning visual and proprioceptive signals inform the brain that the arm is not moving as expected. The aim of mirror visual feedback is to restore the congruity between motor efferences and visual afferences, allowing the subject to unlearn the “learned paralysis” [23]. The effect of the MI could be observed in patients with hemiplegia or another upper limb affectation and in TD subjects [26].

When an individual observes an action, his/her motor system generates an internal representation of the same action, being recruited similarly relative to its execution. This matching mechanism—named mirror mechanism—is thought to be a key substrate of action understanding and imitation [27]. The functional properties of the mirror mechanism indicate that the motor processes and representations that are primarily involved in generating and controlling a given behavior can also be recruited in an individual who is observing someone else displaying that behavior [27]. Thus, mirror neurons fire both when an individual observes an action and when he/she performs a similar action [28]. An observation/execution matching mechanism facilitates the corticospinal pathway, and it is used to improve the motor function; thus, a “mirror box” is a means to facilitate action observation and, therefore, mirror visual feedback is thought to activate the mirror neuron system in a similar way to action observation [29]. Observing an action elicits in the observer’s brain a motor representation of the outcome to which the action is directed, and this motor representation is similar to what would occur if the individual him/herself was planning that action or even just imagining performing it [30,31]. This would allow the individual to identify the goal of the observed action relying mainly on her or his own motor processes and representations [32]. In line with this, in neurological diseases, action observation is thus able to access the motor system, favoring cortical reorganization and ultimately affecting motor abilities [33,34,35]. Such an approach, known as Action Observation Treatment (AOT), has proven effective in improving upper limb motor abilities in several neurological diseases, presenting the advantage of being applicable also at the patient’s own home [35].

Conventional MT is a low-cost, non-invasive therapy that allows upper limb rehabilitation, mostly used in adult stroke patients and, to a lesser extent, in pediatric patients with hemiplegia. It generates benefits in joint position, improves the modulation of the proximal muscles and increases muscle activity, as a result of the recruitment of motor units due to the optical illusion created by the mirror [23,24,25]. MT could be combined with virtual reality, since this therapy has shown upper limb changes (grasp strength, bimanual coordination, etc.) in children with cerebral palsy [36]. Virtual Reality may offer individuals the chance to interact and train with or within interesting and relatively realistic three-dimensional (3D) environments [37]. This allows the intensive repetition of meaningful tasks [38], in a more interesting and autonomous manner than conventional therapy [39]. It is a motivating and entertaining way to engage children in the therapy [19], while enabling the practice and repetition of movements [39,40]. Virtual Reality MT (using immersive glasses) allows the child to integrate into a virtual environment through external devices [37], which, together with mobile applications, represent a great advance in neurorehabilitation, leading to effective and recreational therapies that are easily accessible to the population [11]. It allows improvements in affected upper limb functionality and a greater adherence to therapy by children and families [41,42,43].

The International Classification of Functioning, Disability, and Health (ICF) provides a standard language and framework for the description of health and health-relate states [44]. The ICF emphasizes the importance of measuring or addressing an individual’s function, not only in terms of body structure and function, but also in terms of activities, participation, and environmental factors. Optimal outcome assessment tools should therefore consider the multidimensional nature of function as described by the ICF and measure these multiple facets [45,46]. There is currently a lack of information regarding the measurement tools used for the assessment of different affected upper limb aspects to increase the activity of children with OBP, since most tools assess the range of movement as Mallet scale or goniometric values, based on the reference levels of body structure and function of the ICF [15].

Thus, in the present study, we used the upper limb assessments in terms of activity [46], with the aim of determining the contribution of the application of the Conventional and Virtual Reality mirror therapies to reducing the limitations in activities of daily living related to the affected upper limb use and quality of life in children with OBP between 6 and 12 years of age. The authors hypothesized that Virtual Reality MT would produce higher improvements in the affected upper limb functionality with respect to Conventional MT.

## 2. Materials and Methods

The study was approved by the CEU-San Pablo University Research Ethics Committee, with the approval code: 276/19 (ClinicalTrials.gov Identifier: NCT04412603).

### 2.1. Participants and Recruitment

This was a single-blind (evaluator) study, and the sample was randomized by an external person using EPIDAT™ v.4.2. By convenience sampling, children diagnosed with upper OBP (6–12 years old) were recruited from the ADAYO Association (Spain) and its publications in social networks, as well as through the dissemination of this project among different professionals in the field of health and Centers for Child Development and Early Care from different cities of Spain.

### 2.2. Inclusion and Exclusion Criteria

The inclusion criteria were ages between 6 and 12 years, upper Erb-Duchenne OBP (C5–C6) and extended Erb-Duchenne (C5–C7), preserved functionality to perform the activities, and adequate cognitive level to complete the proposed activities. The exclusion criteria were: associated pathologies, medical complications or cognitive and/or visual impairment that prevented the child from performing the activities, affected upper limb surgeries in the last year, treatment with botulinum toxin in the last three months, the lack of a device with the Android operating system required for the application of Virtual Reality therapy, and no collaborative families and children.

### 2.3. Intervention and Follow-Up

The protocol was elaborated through the comparison of different protocols used in experimental trials, systematic reviews, and meta-analyses on Mirror Therapy and Virtual Reality in children [40,41,42,43,47]. The intervention protocol was performed at home for four weeks, applying Conventional MT or Virtual Reality MT, three days per week, with 20-min each session.

Before initiating the treatment, a meeting was held with the families, where they were informed about the therapies and the protection of their data, and they signed the informed consent; in this meeting, the families were trained in the execution of the therapies, explaining each activity of the protocol to be conducted at home. A video recording was requested, in which the performance of the different exercises with Conventional MT or Virtual Reality MT was shown, in order to modify or adapt the activities prior to the start of the therapy for each participant if required. Neither therapy was initiated until the families were completely confident about the correct performance of each of the activities.

The families were instructed to build the mirror box according to their preferences (to perform Conventional MT), using cardboard pieces of 15 × 15 cm to fit the midline of the trunk; the mirror view was not covered during the exercises. They were also provided with an instructional video. To perform the virtual reality therapy, the families had to download the Virtual Reality MT app for Android: (https://play.google.com/store/apps/details?id=com.sixdimensions.mirrortherapy&hl=es) and acquire the virtual reality glasses in order to perform the proposed exercises (supplementary video to see the execution of both therapies is attached).

The intervention protocol consisted of 6 exercises (3 forearm prone-supination exercises and 3 wrist flexo-extension exercises), performed through the mirror effect, visualizing the non-unaffected upper limb as if it were the affected arm, with Conventional MT or with Virtual Reality MT using the Virtual Reality MT mobile application with a mirror therapy effect (Table 1). Table 2 shows an example of each session per week.

**Figure 1:**
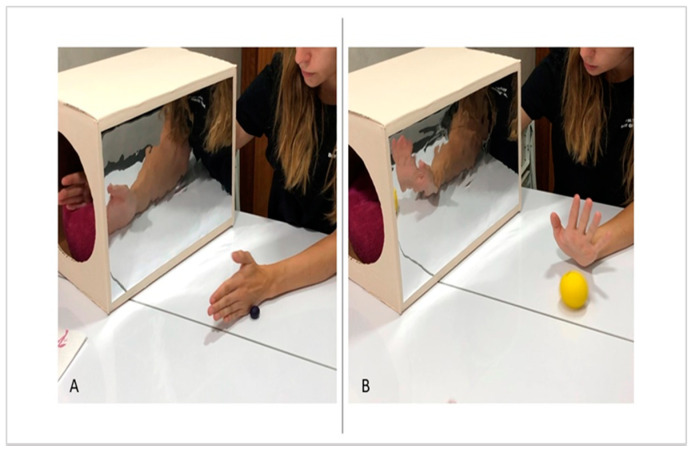
Conventional MT. It is observed the use of mirror in the midline to perform the activities while the child must look in the mirror when the exercise is executed. In the picture (**A**), it is executed the forearm supination to crush the plasticine balls with the back of her left hand (healthy hand), while the right hand is into the box, trying to imitate the same movement. In the picture (**B**), it is seen the wrist extension movement to throw the ball and try to stop it.

Both groups carried out the same exercises with the therapy assigned in each case. In the video S1 can be observed the execution of Conventional MT and Virtual Reality MT. Conventional MT: with the affected limb inside the box, the exercises were performed with the non-affected side while looking at the mirror while the affected side was requested to perform the corresponding movement within its possibilities, depending on the exercise being performed with the healthy upper limb (Figure 1).

**Figure 2:**
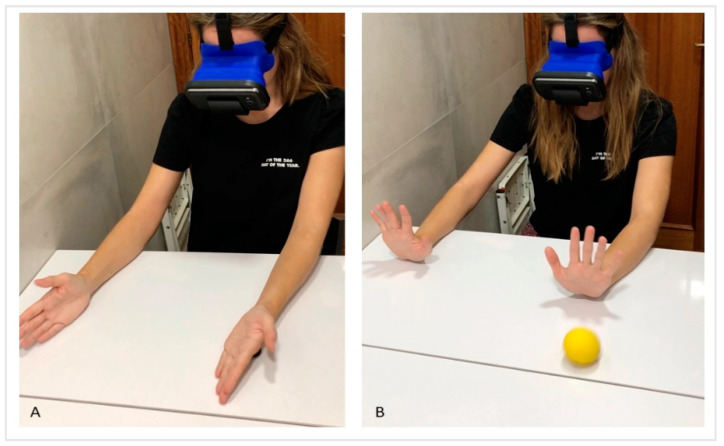
Virtual Reality MT. It is observed the use of virtual reality glasses instead of the mirror box. The child must maintain a straight head position with the glasses in midline, looking towards the hands. The child must execute the request movement with both hands at the same time. The affected hand will perform the activity according to its ability, trying to imitate the proposed movement. In the picture (**A**), it is executed the forearm supination to crush the plasticine balls with the back of the hands. In the picture (**B**), it is seen the wrist extension movement to throw the ball and try to stop it.

Virtual Reality MT: the exercises were performed with the non-affected upper limb, which had to be watched constantly with the virtual reality glasses, in order to interpret that this limb corresponded to the affected side and the affected side accompanied the movement within its possibilities (Figure 2).

The follow-up was carried out each week, recording each session and filling an activity log table to ensure that the therapy was carried out correctly. The families and the therapist conducted an on-line tele-observation of the execution of the activities to prevent possible complications. In addition, the parents were requested to write their opinion regarding the performance of each exercise.

It was estimated that the children in the two treatment groups completed 88.7% of the total dose. Such estimation is based on the fact that, in the study by Ferre et al. [48], where bimanual intensive therapy was carried out at home, and the families received an instruction in the treatment execution and a weekly online follow-up, the participants completed 88.7% of dosage (79.8 h of the total 90 h). Therefore, since the intensity of the therapy was lower in the present study, and given that it was implemented at home with the empowerment of families as in the study of Ferre et al. [48], it was determined that the participants of our study reached the minimum dose, which corresponded to a total of 3 h and 50 min of the 4 h of total dose in both protocols (Conventional MT and Virtual Reality MT).

### 2.4. Outcome Measures

The assessments were performed immediately before starting the therapies (week 0) and after the 4 weeks of therapy (week 4). Both assessments were performed by a blinded researcher.

#### 2.4.1. Primary Measures: Affected Upper Limb Spontaneous Use

The affected upper limb spontaneous use was assessed by the Children’s Hand-use Experience Questionnaire (CHEQ) [49], which was validated for children in the age range of 6 to 18 years with unilateral affectation or disuse of one of their upper limbs; this questionnaire assesses the experience of children using the affected hand in 29 different bimanual activities of daily living [49]. Other criteria evaluated included whether the child used grasp or support when using the affected hand, the effectiveness of the affected upper limb use, the time to perform the activity compared to a child of the same age with typical development, and whether there was some discomfort when performing the activities of the questionnaire. These concepts were assessed on a score scale of 1 to 4, with 1 point representing “least effective” and 4 points corresponding to “most effective” [49].

CHEQ scales are rated on a four-point ordinal scale and raw scores: higher scores indicate a better grasp, less time taken, and greater satisfaction. Previous investigations have shown acceptable unidimensional and high test-retest reliability (ICC 0.87–0.91) in children with unilateral CP; the results indicate a possible ability to detect change [50].

#### 2.4.2. Secondary Measures: Quality of Life

The quality of life was assessed through the Pediatric Quality of Life Inventory Generic Core Scales, PedsQL^TM^ 4.0 [51]. This is a modular instrument for measuring health-related quality of life in children and adolescents aged between 2 and 18 years. It consists of 23 items applicable to healthy school and community populations, as well as pediatric populations with acute and chronic health conditions. It is divided into 4 domains: Physical, Emotional, Social, and School. The questionnaire consists of two questionnaires to assess the perceived quality of life: a questionnaire for the child or adolescent (self-report) and a questionnaire for the parents. Reliability has been previously demonstrated for children (ICC = 0.86) and parents (ICC = 0.89) [51,52,53].

### 2.5. Statistical Analysis

The statistical analysis of the data was performed using SPSS v20.0 for windows (SPSS Inc., Chicago, IL, USA). Given the small sample size, non-parametric analyses were used. The Mann-Whitney U-test to determine the inter-group differences for the variables and the Wilcoxon test for paired samples was performed to compare the before-after treatment differences in the same group for both therapies. Fisher’s exact test was used to determine inter-group differences according to sex and affected upper limb. The results are shown as the median and interquartile range (IQR) with a confidence interval of 95%. The values with *p* < 0.05 were considered statistically significant.

## 3. Results

**Figure 3:**
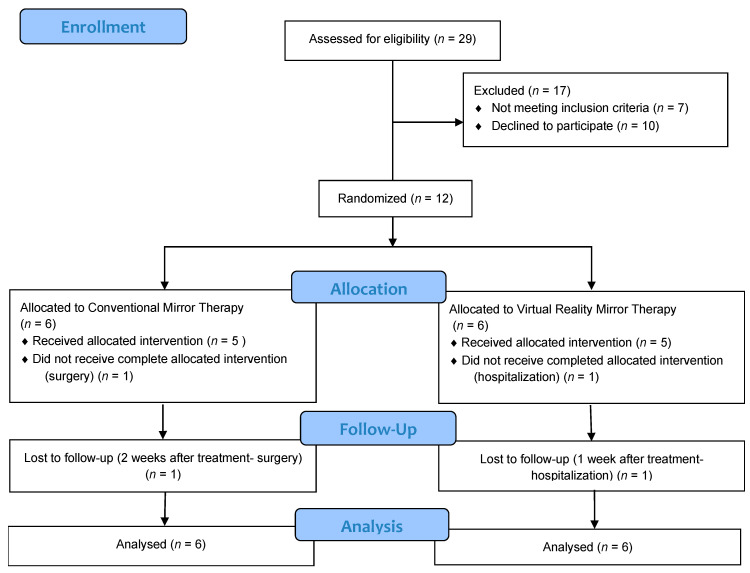
Consort flowchart.

A total of 29 subjects were recruited, of whom 17 were excluded (10 of them did not meet the inclusion criteria and another 7 eventually decided not to participate). The remaining 12 subjects met the inclusion criteria established and were randomly allocated in either of the two intervention groups. Six children were included in the Conventional MT group; however, one child did not receive the complete therapy due to surgery and was lost 2 weeks after the intervention in the follow-up. The other six children were allocated in the Virtual Reality MT group; however, one child did not receive the complete therapy due to hospitalization and was lost 1 week after the intervention in the follow-up. A total of 10 children completed the therapies: five children in the Conventional MT and five children in the Virtual Reality MT (Figure 3).

The mean age of the children was 8.42 years (SD: 3.4). There were no statistically significant inter-group differences) according to age (*p* = 0.9) and sex (*p* = 1.00), although there were differences in the affected upper limb (*p* = 0.04). Fifty percent of the total sample were females, and the other 50% were males. In total, 66.7% of the children had the affectation in the right upper limb, while 33.3% had the affectation in the left upper limb (Table 3).

Table 4 shows the individual characteristics of the participants. Nine children were classified as grade II in the Narakas Classification [9,10]. The affected muscles were flexors and external rotators of shoulder, Biceps Brachii and elbow extensors, forearm supinators, and wrist extensors. The muscle segmental strength (assessed in the affected muscles, as was previously mentioned) was grade 3–4+ in the muscular evaluation [54,55], which means that there was muscular contraction capable of overcoming the resistance imposed by gravity (grade 3) and by an external force (grade 4). Seven participants had been infiltrated with Botulinum toxin- A (BTX-A) one year before the intervention and the most frequently infiltrated muscle was the pectoralis major. The risk factors to acquire OBP considered in this study were: newborn weight >4 kg and instrumentalized delivery.

### 3.1. Primary Results: Spontaneous Use of the Affected Upper Limb (Measured with CHEQ)

No statistically significant inter-group differences were found in any of the variables at week 0 for the spontaneous use of the affected upper limb in the Virtual Reality MT and Conventional MT groups (*p* > 0.05). After applying the treatment (week 4), statistically significant inter-group differences were only obtained for the variable independent tasks (*p* = 0.02) and use of the affected hand with grasp (*p* = 0.04). These results represent statistically significant intra-group differences (pre-post) for Virtual Reality MT group, with a *p* value of 0.02 for independent tasks and a *p* value of 0.04 for the use of the affected hand with grasp. No statistically significant intra-group changes (*p* > 0.05) were seen in the Conventional MT group. (Table 5).

**Figure 4:**
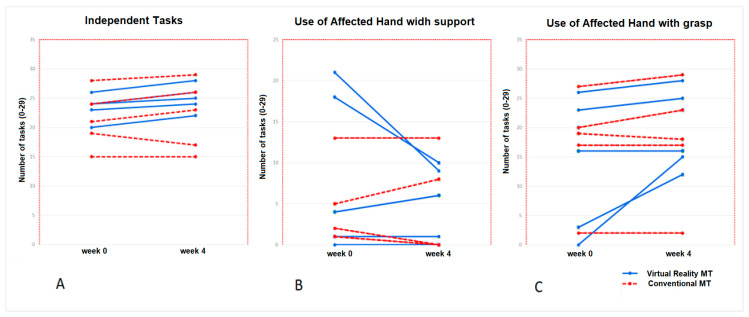
Progression of the number of tasks performed with the participation of the affected hand in Virtual MT and Conventional MT in the ten participants who completed the therapies (pre-post-treatment). Picture (**A**): Independent tasks; it shows an increase in all five children of the Virtual Reality MT group, but not in 2 children of the Conventional MT group. Picture (**B**) shows a great decrease in the use of the affected hand with support in 2 children of the Virtual Reality MT, which would coincide with the increase in the use of the affected hand with grasp for these children in the Virtual Reality MT group (Picture (**C**)).

The affected upper limb use in the baseline situation was present for both the Conventional MT group and the Virtual Reality MT group, shown by the variable “no use AH” with a value of 0 (tasks) for both groups, which was maintained after the intervention. In the Virtual Reality MT group, there was an increase in “use AH with grasp” in 4 activities (*p* = 0.04). There were no changes for this variable in the Conventional MT group (*p* = 0.32), who showed an increase of 3.5 tasks (*p* = 0.41). There was an increase of 2 activities carried out independently (*p* = 0.02) for the Virtual Reality MT group, remaining stable in the conventional MT group (*p* = 0.28). Figure 4 shows the time progression of the ten children who completed the therapies (5 children in the Conventional MT group and 5 children in the Virtual Reality MT group) for the variables related to the use of the affected hand.

**Figure 5:**
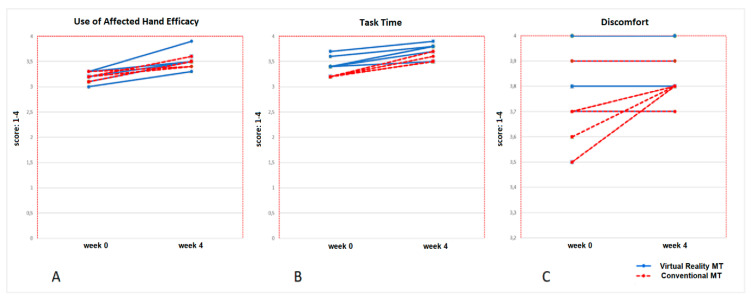
Progression of the use of affected hand efficacy (picture (**A**)), where is observed an increase in all ten children in Virtual Reality MT and Conventional MT and the task time had a similar evolution (picture (**B**)). the score of discomfort does not vary in the five children of the Virtual Reality MT, and a low increase is observed in 3 children of Conventional MT (picture (**C**)).

The efficacy of the use of the affected upper limb obtained a higher increase (0.4 points) in the Virtual Reality MT group with respect to the Conventional MT group (0.2). The task time showed the same increase for both groups (0.2 points) and the discomfort of using the affected upper limb presented no changes for the Virtual Reality MT group, whereas an increase of 0.2 points was obtained by the Conventional MT group. None of the pre-post-therapy changes in these variables were statistically significant (*p* > 0.05). Figure 5 shows the time progression of the ten children who completed the therapies (5 children in the Conventional MT group and 5 children in the Virtual Reality MT group) for the different variables.

### 3.2. Secondary Results: Quality of Life (Measured with PedsQL TM 4.0)

No statistically significant inter-group differences were found at week 0 for any of the variables in the quality of life for the children and parents’ questionnaires in any of the two intervention groups (*p* > 0.05). After applying the treatment (week 4), statistically significant inter-group differences were only obtained in the physical domain variable, answered by the parents (*p* = 0.04), and in the total score of quality of life in the children’s questionnaire (*p* = 0.04). These results represent statistically significant intra-group differences (pre-post) for the Virtual Reality MT group, with a *p* value of 0.03 in the physical domain variable, answered by the parents, and a *p* value of 0.04 for the total score of quality of life in the children’s questionnaire. No statistically significant intra-group changes (*p* >0.05) were detected in the Conventional MT group (Table 6).

**Figure 6:**
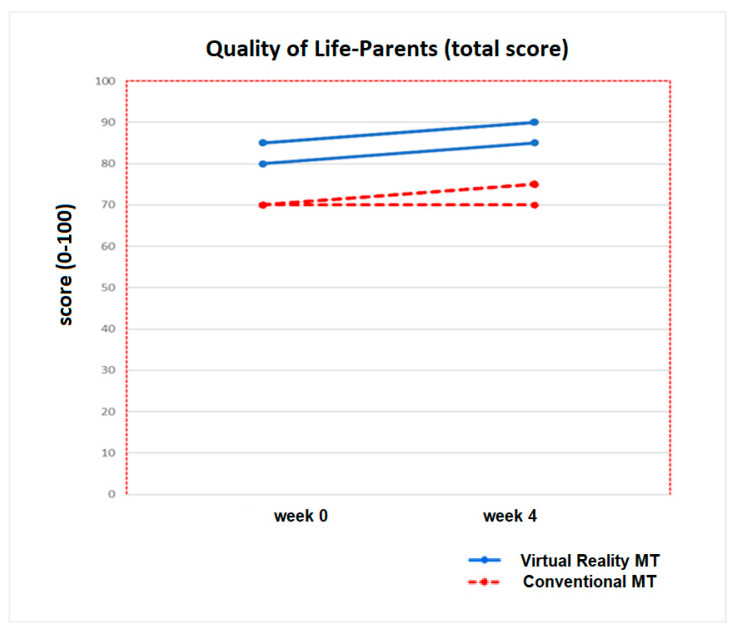
Progression of the Quality of life- Parents (total score) of the ten children who completed both therapies (Virtual Reality MT: 5 children and Conventional MT: 5 children).

No statistically significant differences (*p* > 0.05) were found for the total score of the quality of life questionnaire for parents, whose increase was 2.2 points for the Virtual Reality MT group and 0.7 points for the Conventional MT group (Figure 6).

**Figure 7:**
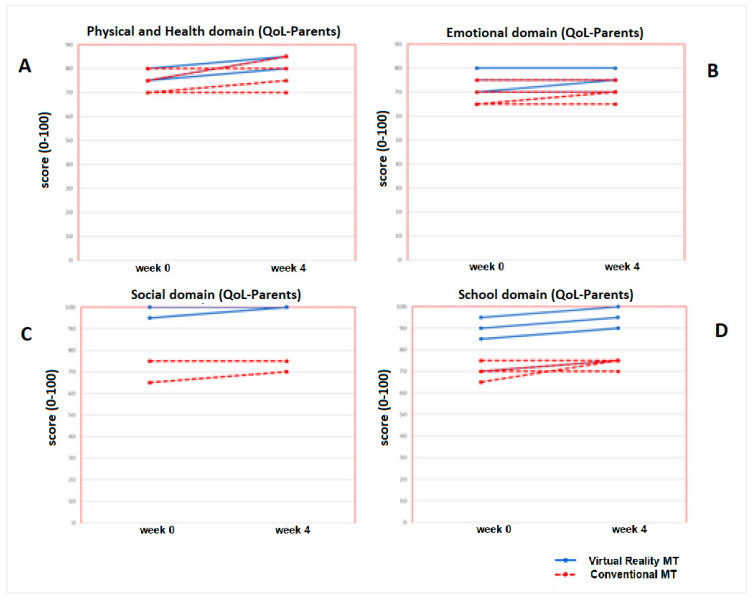
Progression of Quality of Life-Parents (domains) in the five children of the Virtual Reality MT and the five children who performed Conventional MT. In the picture (**A**), it can be seen the progression of the Physical and Health domain and in the picture (**B**), the Emotional domain evolution. In the picture (**C**), the progression of the Social domain is represented and the picture (**D**), the school domain evolution.

The increase in the Conventional MT group was 1 point (*p* = 0.14). No significant changes (*p* > 0.05) were obtained for any of the other the variables (EmD (byP), SocD (byP), SchD (byP)) or any of the categories of the parents’ questionnaire after the treatment (week 4). Figure 7 shows the domains progression of Virtual Reality MT and Conventional MT of the ten children who completed both therapies.

**Figure 8:**
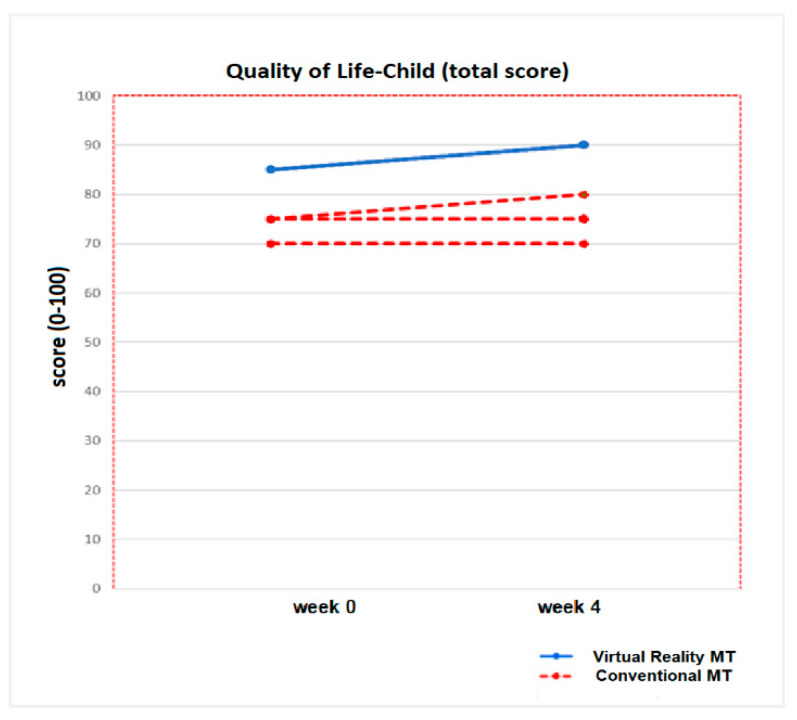
Progression of the Quality of life- Child (total score) of the ten children who completed both therapies (Virtual Reality MT: 5 children and Conventional MT: 5 children).

The total score in the questionnaire answered by the children showed a post-treatment increase of 3.5 points (*p* = 0.04) for the Virtual Reality MT group and 2 points for the Conventional MT group (*p* = 0.07). Figure 8 shows the progression of the Quality of life-Child (total score) of the ten children who completed both therapies.

**Figure 9:**
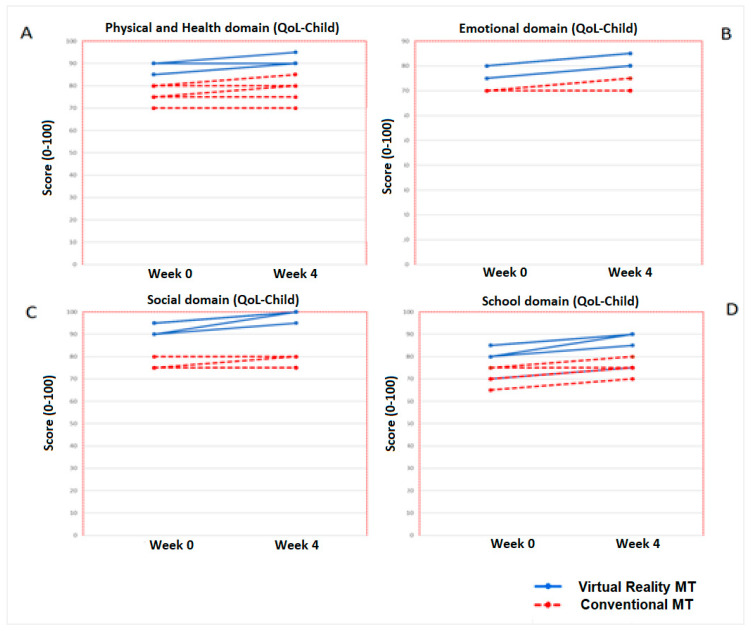
Progression of the Quality of Life-Child (domains) in the five children of the Virtual Reality MT and the five children who performed Conventional MT. In the picture (**A**), it can be seen the progression of the Physical and Health domain. The picture (**B**) shows the Emotional domain evolution, the picture (**C**), the progression of the Social domain and the progression of School domain is represented in the picture (**D**).

The greatest change in the variables that make up the questionnaire occurred in “Physical Domain” (as in the parents’ questionnaire), with a non-statistically significant increase of 4.7 points (*p* = 0.07), whereas the rest of the variables (EmD (by Ch), SocD (by Ch) and SchD (by Ch)) showed a post-treatment increase of 2.5 points without statistical or clinical significance (Figure 9).

Once the registration tables sent by the families in each of the weekly follow-ups were analyzed, it was verified that all the children in the Virtual Reality MT and Conventional MT groups completed the intervention time of 20 min per day, performing the total therapy dose (4 h).

## 4. Discussion

An adequate bimanual functional performance and emotional and physical well-being linked to the quality of life promote the independence and participation of the child with affected upper limb in the execution of activities of daily living. Thus, it is necessary to know how the usual activities for the child are carried out in their lives and whether the activities are achieved with the use of the healthy hand or with both hands. In addition, the perceived experience of hand use by children and their parents should be identified, in order to establish individual interventions to reduce limitations in bimanual activities and promote their satisfaction in the execution [49].

This study is the first to research the influence of Conventional MT and Virtual Reality MT on OBP, with an increase of functionality being observed after 4 weeks of therapy in the affected upper limb, measured with the CHEQ questionnaire for the Virtual Reality MT group, in terms of independently performed tasks (increase of 2 tasks) and use of affected hand with grasp (increase of 4 tasks). However, despite the improvements observed in the Virtual Reality group, the task execution time did not decrease significantly and the efficacy of the use of the affected hand, as well as the discomfort of use, remained stable, which could mean that, in spite of using the affected hand in an increased number of tasks performed independently and with grasp. There may not be a parallel progression between improving independently performed tasks and the efficacy of the use of the affected hand with discomfort, since children continue to feel uncomfortable when the affected hand is involved. In the Conventional MT group, the changes were maintained after therapy, although an increase of 3.5 tasks was obtained in the use of the affected hand with support. As in the Virtual Reality MT group, there was no parallel progression between improving the efficacy of the use of the affected hand with support and discomfort. This could be due to the fact that children rely on extrinsic motivation to perform the tasks. That is, they are driven by a result from completing the tasks through fun and games, with a reward that is appropriate to their objectives, sometimes receiving negative feedback from the use of the affected hand in the bimanual task [56,57]. This may be caused by alterations in motor functions and poor motivation in the use of the affected hand due to the present movement restrictions and the lack of cortical information [58].

The scientific evidence shows statistically significant results in the groups treated with virtual reality, leading to considerable changes in the affected upper limb functionality [40,59,60,61,62]. As is observed in a previous study about the effect of Virtual Reality compared to conventional physiotherapy on upper limb function in children with OBP [40], Virtual Reality Mirror Therapy combined with Armeo Spring™ Pediatric (Hocoma AG, Switzerland) resulted in improvements at the level of mobility and affected upper limb strength, obtaining higher scores on the Mallet scale, being significantly more effective than conventional therapy and achieving the main objective of restoring basic functionality to the affected upper limb abilities. Virtual reality has also been used for the rehabilitation of activities of daily living using a virtual environment, obtaining a positive effect as a complementary tool to conventional rehabilitation [60]. The results obtained in this study, that is, the improvements in functionality (independent tasks and the use of the affected hand with grasp) assessed with the CHEQ questionnaire [49,50], could be transferred to certain activities of daily living, such as personal hygiene, leisure, and sports activities. This would reduce activity limitations and participation restrictions, promoting greater independence in their daily environment with their peers with typical and atypical development.

In the study of Majnemer et al. [63], the quality of life perceived by children with CP was compared with the parents’ perception using the PedsQL scale. The results showed a lower score in the questionnaire for the parents, observing a difference in the perceived quality of life between children and parents, which suggests that the parents tend to underestimate the child’s quality of life [63]. Similarly, in the present study, there was an increase (3.5 points) in the quality of life in the children’s report for the Virtual Reality MT group with respect to the parents’ report (2.2 points). These data also show clinically relevant gains in the quality of life for children with upper OBP, which led to an integration of the functional gains obtained in the child’s everyday life. In the Conventional MT group, only two families reported a decrease in pain; the rest of the families did not obtain relevant changes in any of the assessed aspects. It has been documented in the quality of life measurement of children with chronic health conditions that the information provided by the parents was not equivalent to that reported by the children [64,65]. The findings indicate that parents’ reports cannot be replaced with children’s self-reports [66]. In the same group (Virtual Reality MT), the highest increase for the questionnaires answered by parents and children, with significant changes for the parents’ report, occurred in the physical domain, which could mean that, among the domains of quality of life, this domain would be the most objective in the evaluation and, therefore, the one in which both the parents’ and children’s reports could obtain a similar score.

In the Conventional MT group, the values did not obtain significant changes for either the parents’ reports or the children’s reports, with higher increases in the children’s reports. The greatest changes in the children’s reports were in the social and school domains, which could mean that these children would feel safer and perceive greater well-being in their natural environment, although these changes were not clinically relevant.

The perceptions of the families and children were very positive, since they adhered to the therapy, the proposed times were completed, the structured activities were conducted correctly, and the children were motivated to carry out the exercises. The children from the Virtual Reality MT group did not want the intervention to end, as they played an entertaining role. The children in the Conventional MT group reported that it was difficult to keep the attention at the end of the therapy, although they found it amazing to discover in the visual reflex that their hand “influenced” the correct execution of the exercises, which made them want to achieve the goal of completing the exercises. The main interest of all children and families was to promote the participation of the affected upper limb in their everyday life, and they all claimed to be satisfied after the intervention, i.e., both the Virtual Reality MT and Conventional MT groups.

The Virtual Reality MT and Conventional MT families reported that the implementation of the intervention at home was easy, that they were satisfied with the achievements and that it allowed them to see the capacities and not only the limitations of their children in some activities of daily living, improving the family-child-therapist relationship. This concept means that the family is a key component of the child’s environment and the relationship with the therapist (through follow-up) can be used as the context to deliver critical components (i.e., intensity, repetition, feedback) of established therapies with the child [67]. Hadders-Algra et al. [68] state that a family-centered approach creates a richer and more varied array of opportunities by coaching the family to encourage the child to use the affected upper limb in the usual environment, improving the quality of life in the execution of bimanual activities of daily living: eating, personal hygiene, dressing-undressing…, etc. Therapist-family-child feedback was maintained within a weekly family-centered program, where the monitoring table, the activities, the doubts of the families, the complications, the manifestations of the children about the activities or changes to promote their motivation following their preferences were reviewed. In this way, adherence to the treatment, continuity, and compliance with the proposed dose and activities were promoted. Thus, the intervention at home directed by the family and supervised by the therapist would be successful in the improvement of the affected upper limb functionality.

The limitations of the study were the lack of further scientific evidence for Virtual Reality MT and Conventional MT to compare the results obtained in the present study, the absence of objective outcomes, and the small sample size, which do not allow generalizing the findings to the OBP population, since this is considered a pilot study. Future studies should use a larger sample size and create a randomized controlled trial applying Virtual Reality MT and Conventional MT with different treatment doses, to study possible improvements with more dose. In addition, objective outcomes should be included, with measurement tools in all the domains (structure, function, activity, and participation) of ICF to improve the quality of future work in this research line and the empowerment of OBP families at home, with the aim of increasing the children’s and families’ participation in the natural environment and favoring the relationship between them.

## 5. Conclusions

Compared to conventional MT, Virtual Reality MT would be a home-based therapeutic complement to increase independent bimanual tasks using grasp in the affected upper limb and improve the quality of life for children diagnosed with upper OBP in the age range of 6–12 years.

## Figures and Tables

**Table 1:** Exercises to perform for 4 weeks in both therapies.

**Forearm Prone-Supination Exercises**	
**Sound Bottle**	-Material: fill a bottle with beads, chickpeas, lentils, or grains of rice, stick chopsticks so that it takes longer to move the inner contents/rain stick. -Exercise: make forearm prono-supination so that the content falls from one side to the other inside the bottle. 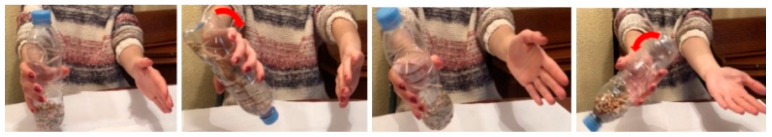
**Cookies**	-Material: small plasticine balls. -Exercise: with your forearm resting on the table, from a neutral prone-supination position, make supination to crush the plasticine balls with the back of your hand. 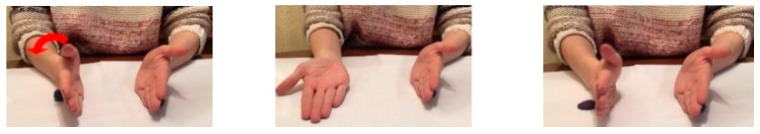
**The Tower**	-Material: small plasticine balls. -Exercise: with your forearm in supination resting on the table, gradually place the plasticine balls on the palm of your hand to form a tower. 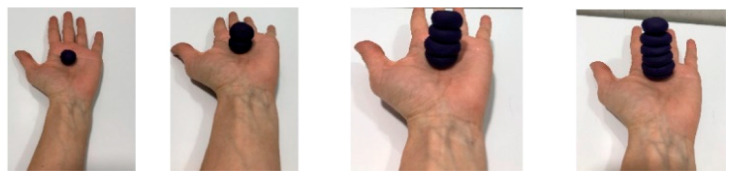
**Flexo-Extension Wrist Exercises**	
**Ball Wheel**	-Material: small ball, i.e.,: tennis ball. -Exercise: with your forearm resting on the table, slowly throw the ball and try to stop it by making wrist extension. 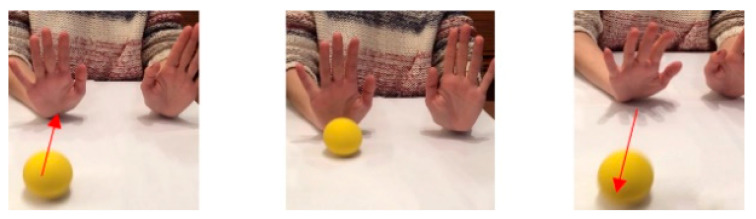
**Doughnuts**	-Material: make small plasticine rings. -Exercise: with your forearm resting on the table, do wrist extension and place the doughnuts on your fingers while keeping your wrist in extension. You can stop and rest when you are tired. 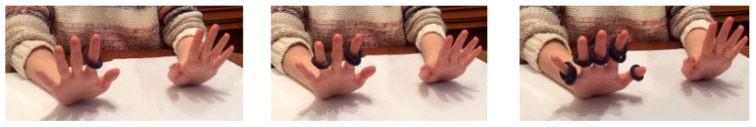
**The Marble**	-Material: a small ball of plasticine. -Exercise: put a small ball on the back of your hand and make wrist extension to keep the ball from falling (move the small ball on the back of your hand). 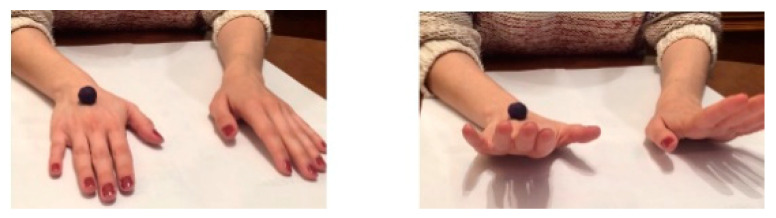

**Table 2:** Examples of each session per week for 4 weeks of treatment (Conventional Mirror Therapy (MT) or Virtual Reality MT).

Protocol: 4 Weeks Dose: 20 min per/day	Day 1	Day 2	Day 3
5 min (morning)	Forearm Prone-supination 1-Sound Bottle	Wrist flexion-extension 5-Doughnuts	Forearm Prono-supination 3-Tower
5 min (morning)	Forearm Prone-supination 2-Cookies	Wrist flexion-extension 6-The Marble	Wrist flexion-extension 4-Ball Wheel
5 min (afternoon)	Forearm Prone-supination 3-Tower	Forearm Prone-supination 1-Sound Bottle.	Wrist flexion-extension 5-Doughnuts
5 min (afternoon)	Wrist flexion-extension 4- Ball Wheel	Forearm Prone-supination 2-Cookies	Wrist flexion-extension 6-The Marble

**Table 3:** Characteristics of the variables in the Virtual Reality MT and Conventional MT groups.

VARIABLES	Total (*n* = 12)	Virtual Reality MT (*n* = 6)	Conventional MT (*n* = 6)	*p*-Value
AGE years, mean (SD)	8.42 (3.4)	8.5 (3.5)	8.4 (3.3)	0.9
SEX				
Male. Num (%)	6 (50)	3 (50)	3 (50)	1.00
Female. Num (%)	6 (50)	3 (50)	3 (50)	
AFFECTED UPPER LIMB				
Left. Num (%)	4 (33.3)	1 (16.7)	3 (50)	0.04 *
Right. Num (%)	8 (66.7)	5 (83.3)	3 (50)	

* Statistically significant inter-group differences when *p* value < 0.05 (Age: Mann-Whitney U-test; Sex and Affected upper limb: Fisher’s exact test).

**Table 4:** Individual characteristics of the children.

Ch	Age	Sex	Injured B.P. Roots	Aff. UL	Aff. Muscles/m.S.S	Narakas Classf.	Risk Factor PBO	BTX-A	Performed Therapy
**1**	12	F	C5–C6	R	-Shoulder: Flexors: 4− Ext.rot: 4− -Elbow: Biceps and Extensors: 3+/3 -Forearm: Supinators: 3+	I	>4 kg born	Pectoralis major Biceps brachii	Virtual Reality MT
**2**	11	M	C5–C6	R	-Shoulder: Flexors: 4 Ext.rot: 4 -Elbow: Bicepsand extensors: 4/3+ -Forearm: Supinators: 3+	I	>4 kg born	Pectoralis major Biceps brachii	Conventional MT
**3**	8	F	C5–C6–C7	R	-Shoulder: Flexors: 4− Ext.rot: 4 -Elbow: Biceps and extensors: 4/3+ -Forearm: Supinators: 3+ -Wrist: Extensors: 4	II	>4 kg born	Pectoralis major Biceps brachii	Conventional MT
**4**	7	F	C5–C6	L	-Shoulder: Flexors: 4 Ext.rot: 3+ -Elbow: Biceps and extensors: 3+/3+ -Forearm: Supinators: 3+	I	Instrument. delivery	No	Conventional MT
**5**	7	M	C5–C6–C7	R	-Shoulder: Flexors: 4− Ext.rot: 3+ -Elbow: Biceps and extensors: 4/3+ -Forearm: Supinators: 3+ -Wrist: Extensors: 4	II	Instrument. delivery	No	Virtual Reality MT
**6**	9	M	C5–C6–C7	L	-Shoulder: Flexors: 4 Ext.rot: 4 -Elbow: Biceps and extensors: 4−/3+ -Forearm: Supinators: 3+ -Wrist: Extensors: 4	II	>4 kg born	No	Conventional MT
**7**	6	F	C5–C6–C7	R	-Shoulder: Flexors: 4 Ext.rot: 3+ -Elbow: Biceps and extensors: 4/3+ -Forearm: Supinators: 3+ -Wrist: Extensors: 4	II	Instrument. delivery	Biceps brachii Pronators	Conventional MT
**8**	8	M	C5–C6–C7	R	-Shoulder: Flexors: 4 Ext.rot: 4 -Elbow: Biceps and extensors: 4/4 -Forearm: Supinators: 4 -Wrist: Extensors: 4	II	Instrument. delivery	Pectoralis major	Virtual Reality MT
**9**	8	F	C5–C6–C7	L	-Shoulder: Flexors: 4 Ext.rot: 3+ -Elbow: Biceps and extensors: 3+/3+ -Forearm: Supinators: 3+ -Wrist: Extensors: 4	II	Instrument. delivery	Biceps brachii pronators	Virtual Reality MT
**10**	10	M	C4–C5–C6–C7	R	-Shoulder: Flexors: 4− Ext.rot: 4− -Elbow: Biceps and extensors: 3+/3+ -Forearm: Supinators: 3+ -Wrist: Extensors: 3+	II	>4 kg born	No	Virtual Reality MT
* 11	6	F	C5–C6–C7	R	-Shoulder: Flexors: 4 Ext.rot: 4 -Elbow: Biceps and extensors: 4/3+ -Forearm: Supinators: 3+ -Wrist: Extensors: 4−	II	Instrument. delivery	No	Virtual Reality MT
* 12	9	M	C5–C6–C7	L	-Shoulder: Flexors: 4 Ext.rot: 3+ -Elbow: Biceps and extensors: 4/3+ -Forearm: Supinators: 3+ -Wrist: Extensors: 4−	II	Instrument. delivery	Pectoralis major	Conventional MT

Age expressed in years. Sex: F: Female, M: Male; Injured B.P. roots: Injured Brachial Plexus roots; Aff. UL: Affected Upper Limb, R: right; L: left; Aff.muscles: Affected muscles; m. S. S: muscular segmental strength; Narakas Classif: Narakas Classification; Risk Factor OBP; Instrument.delivery: Instrumentalized delivery; Conventional MT: Conventional Mirror Therapy; Virtual Reality MT: Virtual Reality Mirror Therapy. * Did not complete the therapy and follow-up.

**Table 5:** Results of spontaneous use for Conventional MT and Virtual Reality MT.

Spontaneous USe	Total Sample (*n* = 12)	^+^*p* Value	Virtual Reality MT (*n* = 6)	*p* Value	Conventional MT (*n* = 6)	*p* Value
Indep. Task						
Week 0	23.5 (15, 28)	0.12	24 (21, 28)		20 (15, 27)	
Week 4	24.5 (15, 29)	0.02 *	26 (23, 29)	0.02 *	19.5 (15, 24)	0.78
No indep. Task						
Week 0	2 (0, 9)	0.10	1 (0, 8)		4 (2, 9)	
Week 4	2.5 (0, 7)	0.19	0.5 (0, 5)	0.18	5 (0, 7)	0.28
No rel. Task						
Week 0	2.5 (0, 8)	0.75	5 (0, 7)		2 (0, 8)	
Week 4	1.5 (0, 8)	0.14	3 (0, 3)	0.13	6.5 (0, 8)	0.28
Non-use AH						
Week 0	0 (0, 3)	0.90	0 (0, 3)		0 (0, 2)	
Week 4	0 (0, 3)	0.41	0 (0, 3)	1.00	0 (0, 0)	0.32
Use AH support						
Week 0	2 (0, 21)	0.37	1 (0, 18)		4 (0, 21)	
Week 4	3.5 (0, 13)	0.32	0.5 (0, 10)	0.46	7.5 (0, 13)	0. 41
Use AH grasp						
Week 0	19 (0, 27)	0.14	20 (3, 27)		16 (0, 27)	
Week 4	17.5 (0, 29)	0.04 *	24 (12, 29)	0.04 *	15.5 (2, 17)	0.32
Efficacy						
Week 0	3.3 (2,3, 4)	0.74	3.2 (2.3, 3.8)		3.3 (3.1, 4)	
Week 4	3.5 (2.3, 4)	0.59	3.7 (2.3, 4)	0.79	3.5 (2.9, 4)	0.70
Task time						
Week 0	3.4 (1.9, 4)	0.19	3.5 (3.2, 4)		3.3 (1.9, 4)	
Week 4	3.7 (1.7, 4)	0.39	3.8 (3.1, 4)	0.58	3.6 (1.7, 4)	0.45
Disconfort						
Week 0	3.9 (2.9, 4)	0.55	3.9 (3.6, 4)		3.7 (2.9, 4)	
Week 4	3.9 (2.9, 4)	0.17	3.9 (3.5, 4)	0.85	3.9 (2.9, 4)	0.32

Virtual Reality MT: Virtual Reality Mirror Therapy; Conventional MT: Conventional Mirror Therapy; Indep. Task: Independent Tasks; No Independ. Tasks: No independent tasks; No rel. Tasks: No relevant tasks; No use AH: No use of Affected Hand; Use AH: Use of the Affected Hand; Use AH with support: Use of the Affected Hand with support; Use AH with grasp: Use of the Affected Hand with grasp. Independent task, no-independent task, no relevant task, non-use AH, Use AH with support, and use AH with grasp are expressed in total number of tasks. Efficacy, task time and discomfort are expressed in a 4-point scale (1–4; 1: lowest score, 4: highest score). Values are expressed in median (interquartile range; (IQR). ^+^ Statistically Significant Difference (intergroup difference assessed with Mann-Whitney U-test) when *p* value < 0.05. * Statistically Significant Difference (pre-post intragroup difference assessed with Wilcoxon’s test) when *p* value < 0.05.

**Table 6:** Results of quality of life for Conventional MT and Virtual Reality MT.

Quality of Life	Total Sample (*n* = 12)	*p* Value	Virtual Reality MT (*n* = 6)	*p* Value	Conventional MT (*n* = 6)	*p* Value
PhysD (Ch)						
Week 0	76.6 (50, 100)	0.29	87.5 (53.1, 96.9)	0.07	75 (50, 100)	0.18
Week 4	85.9 (56.2, 96.9)	0.16	92.2 (56.2, 96.9)		75 (56.2, 93.7)	
PhysD (P)						
Week 0	74.1 (46.9, 96.9)	0.57	75 (46.9, 96.9)		72.9 (59.4, 93.7)	
Week 4	81.2 (65.6, 100)	0.04 *	82.8 (71.9, 100)	0.03^+^	73.9 (65.6, 90.6)	0.14
EmD (Ch)						
Week 0	71.5 (50,100)	0.45	80 (45, 100)		60 (60, 100)	
Week 4	74.5 (45, 100)	0.62	82.5 (50, 100)	0.46	65 (90, 50)	0.28
EmD (P)						
Week 0	75 (50, 100)	0.37	75 (65, 100)		65 (50, 80)	
Week 4	75 (50, 100)	0.08	75 (65, 100)	0.59	65 (50, 70)	0.41
SocD (Ch)						
Week 0	90 (40, 100)	0.06	95 (70, 100)		70 (40, 90)	
Week 4	90 (40, 100)	0.06	97.5 (80, 100)	0.1	77.5 (40, 90)	0.11
SocD (P)						
Week 0	87.5 (55, 100)	0.06	100 (55, 100)		70 (60, 90)	
Week 4	90 (60, 100)	0.07	100 (90, 100)	0.28	70 (60, 90)	0.1
SchD (Ch)						
Week 0	75 (60, 100)	0.06	82.5 (65, 95)		65 (60, 80)	
Week 4	80 (50, 95)	0.2	85 (60, 100)	0.52	72.5 (50, 90)	0.32
SchD (P)						
Week 0	77.6 (45, 100)	0.12	85 (45, 100)		70 (55, 70)	
Week 4	85 (60, 95)	0.23	87.5 (75, 95)	0.07	71.5 (60, 90)	0.1
Total QoL (Ch)						
Week 0	81 (52.5, 96.7)	0.08	84.2 (59.5, 96.7)		71.2 (52.5, 85)	
Week 4	85.2 (59, 96.5)	0.04 *	87.7 (62.8, 95.5)	0.04 *	73.2 (42.5, 95)	0.07
Total QoL (P)						
Week 0	78.9 (57.3, 98.4)	0.14	82.8 (60.5, 98.4)		70.5 (57.3, 83.4)	
Week 4	81.2 (58.9, 91.9)	0.09	85 (45, 100)	0.12	71.2 (60.3, 85.4)	0.07

Virtual Reality MT: Virtual Reality Mirror Therapy; Conventional MT: Conventional Mirror Therapy; PhysD (by Ch)/(by P): Physical Domain (answered by the children)/(answered by the parents); EmD: Emotion Domain; Soc.D: Social Domain; schD: School Domain; All categ: All categories. The results are expressed in a frequency scale (0–100). Pre: pre-therapy; Post: post-therapy. The pre-post values are expressed in median (interquartile range; IQR). * Statistically Significant Differences (bilateral asymptotic significance in Wilcoxon’s test: non-parametric test) when *p*-value < 0.05.

## Supplementary Materials

The following are available online at https://www.mdpi.com/2077-0383/9/9/3021/s1, Video S1: Execution of Conventional MT and Virtual Reality MT.
